# Double-Outlet Right Ventricle, Pulmonary Atresia, and Discontinuous Branch Pulmonary Arteries Supplied by Bilateral Ducti

**DOI:** 10.1016/j.jaccas.2021.04.020

**Published:** 2021-06-23

**Authors:** Kamel Shibbani, Bijoy Thattaliyath, Michael Bunker, Shafkat Anwar, Ravi Ashwath

**Affiliations:** a Division of Pediatric Cardiology, University of Iowa Stead Family Children’s Hospital, Iowa City, Iowa, USA; b University of California-San Francisco, Center for Advanced 3D+ Technologies, San Francisco, California, USA; c Division of Cardiology, Department of Pediatrics, University of California-San Francisco, School of Medicine, San Francisco, California, USA

**Keywords:** 3D model, bilateral ductus arteriosus, double-outlet right ventricle, hypoplastic left heart, pulmonary atresia, right aortic arch, CTA, computed tomographic angiography, DORV, double-outlet right ventricle, PA, pulmonary artery, SVC, superior vena cava, TAPVC, total anomalous pulmonary venous connection

## Abstract

We present a rare case of double-outlet right ventricle with pulmonary atresia and discontinuous branch pulmonary arteries supplied by bilateral ducti from a right aortic arch. To our knowledge, this is only the second documented case of double-outlet right ventricle with bilateral ducti. (**Level of Difficulty: Advanced.**)

## Central Illustration

**Figure:**
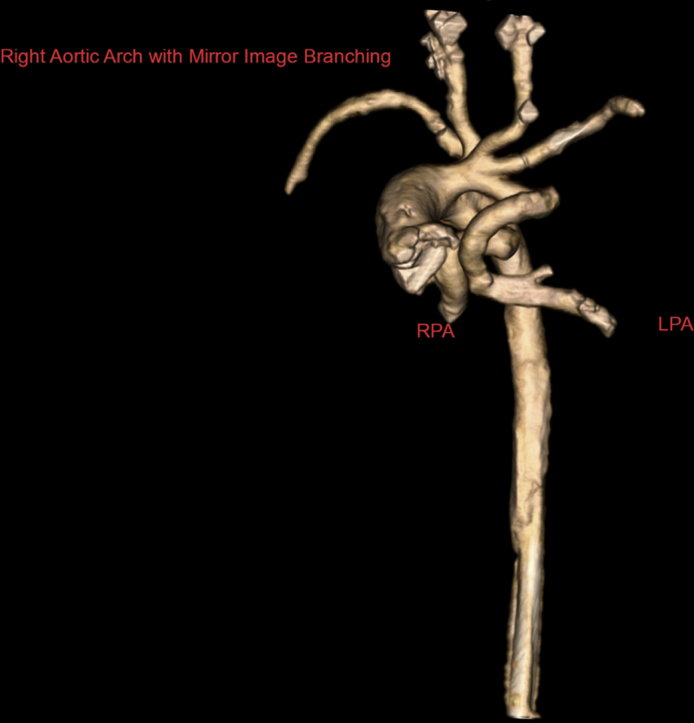

## History of Presentation

A 30-year-old woman was referred for fetal echocardiography that demonstrated a fetus with a hypoplastic left ventricle and a hypoplastic mitral valve, a single outflow tract identified as the aorta arising from the right ventricle, pulmonary atresia with absence of the main pulmonary artery (PA), and poorly visualized branch PAs and pulmonary veins.

**Learning Objectives**

• To create a differential diagnosis for congenital heart disease with a single outflow tract.
• To understand the clinical implication of identifying the nature and source of pulmonary blood flow.
• To understand the association between pulmonary atresia and bilateral ducti.

## Medical History

Our patient was a stable, full-term infant with the prenatal fetal echocardiographic findings just described. She was born at 38 weeks’ gestation and was immediately started on prostaglandin E given the findings of fetal echocardiography.

## Differential Diagnosis

The typical differential diagnosis for a single outflow tract includes truncus arteriosus and pulmonary atresia with and without ventricular septal defect. Less common diagnoses include double-outlet right ventricle (DORV) with pulmonary atresia or aortic atresia, among others.

When such a differential is present, identification of the source of pulmonary blood flow (aortopulmonary collateral vs patent ductus arteriosus) is paramount as it dictates the immediate need for intervention in ductal dependent lesions and planned future intervention in case of aortopulmonary collaterals.

## Investigations

In our patient, post-natal transthoracic echocardiography showed DORV, mitral atresia, pulmonary atresia with primum and secundum atrial septal defects, hypoplastic left ventricle, and an unrestrictive ventricular septal defect. A right aortic arch was noted to arise from the right ventricle, and there were discontinuous PAs supplied by vessels arising from the aorta. There were bilateral superior venae cava (SVC), with the right SVC draining into the right atrium and the left SVC draining directly into the left-sided atrium.

**Figure 1:**
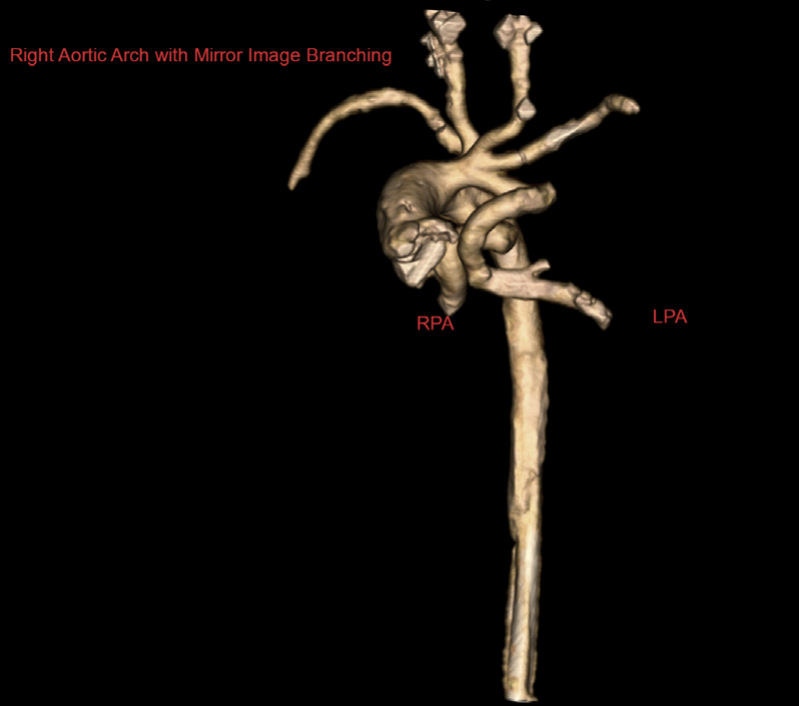
Volume-Rendered Computed Tomographic Reconstruction Showing a Right Aortic Arch With Mirror Image Branching and Discontinuous Pulmonary Arteries The left pulmonary artery (LPA) is seen originating from the undersurface of the left innominate artery. RPA = right pulmonary artery.

**Figure 2:**
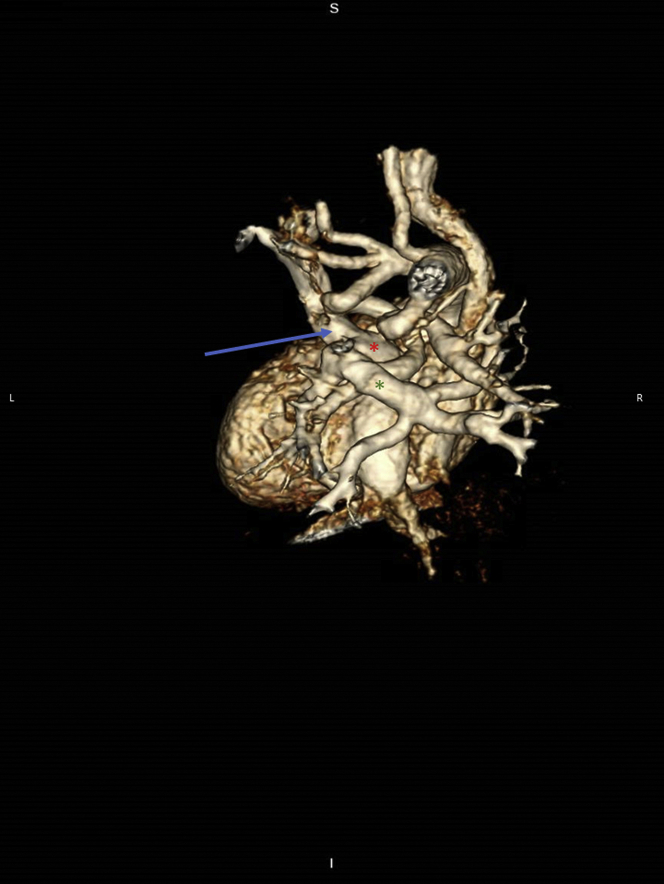
Total Anomalous Pulmonary Venous Connection With Drainage Into the Junction of the Left-Sided Superior Vena Cava and the Left-Sided Atria **Blue arrow** indicates the left superior vena cava. **Red asterisk** indicates the left-sided atrium. **Green asterisk** indicates the venous confluence into which the pulmonary veins drain. This in turn drains into the left-sided atrium.

**Figure 3:**
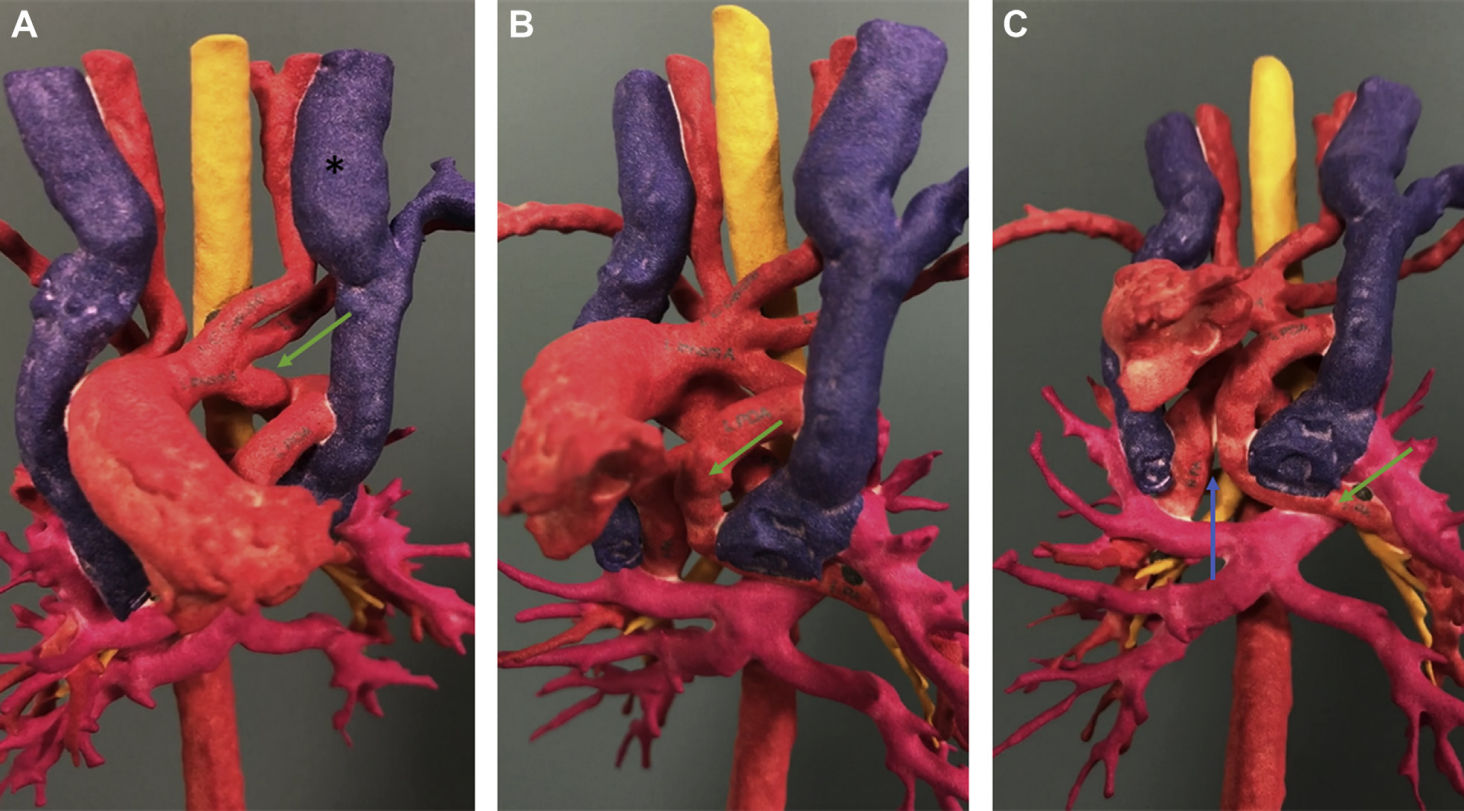
Three-Dimensional Model Showing the Course of the Left Ductus Arteriosus The **green arrow** highlights the take-off from the undersurface of the left innominate artery **(A)** and its course **(B)** as it continues to become the left pulmonary artery. The discontinuous nature of the left and right pulmonary arteries is highlighted by a **blue arrow (C)**. The left-sided superior vena cava is indicated by a **black asterisk (A)**.

**Figure 4:**
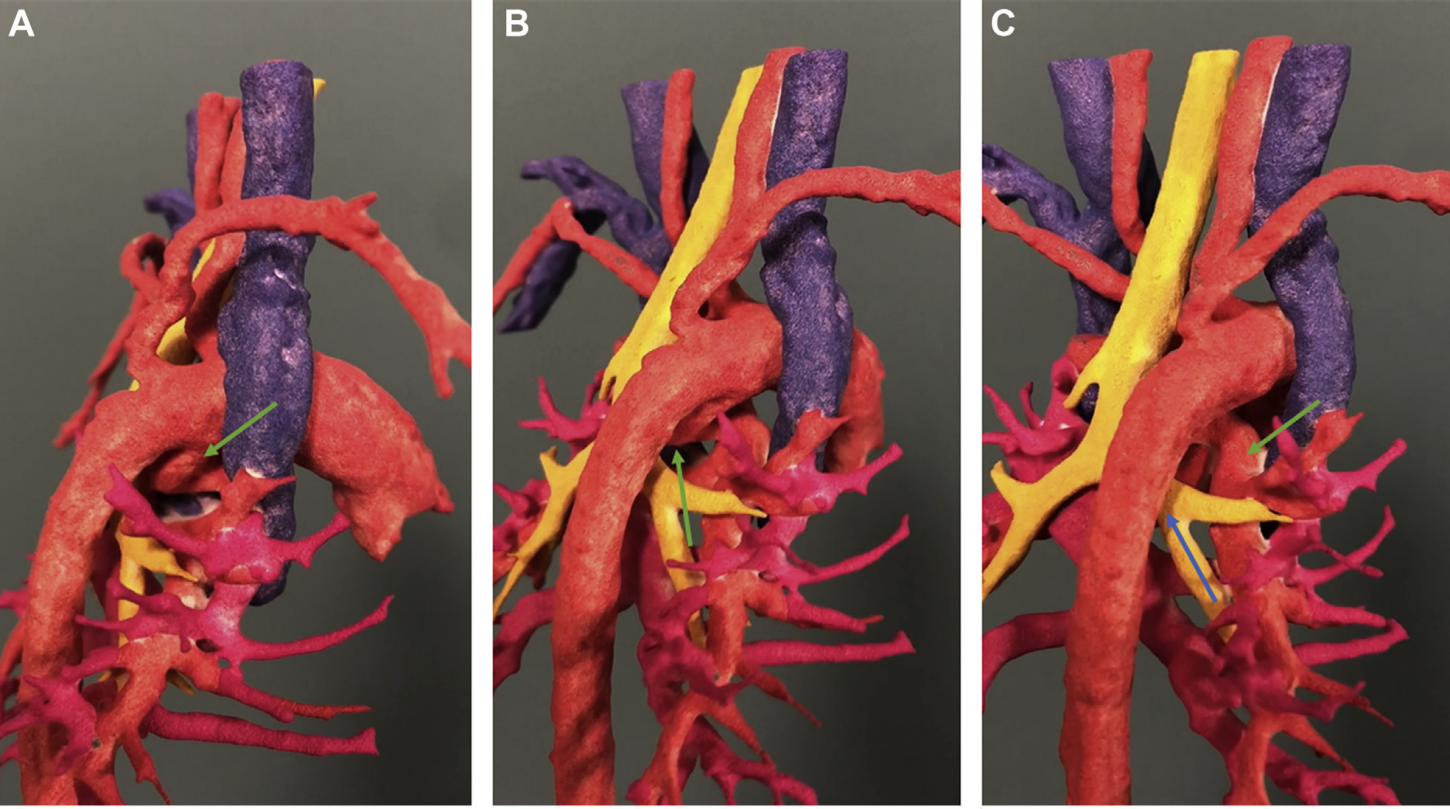
Three-Dimensional Model Showing the Course of the Right Ductus Arteriosus The **green arrow** highlights the take-off from the underside of the right aortic arch **(A)**, showing the ductus taking a tortuous course **(B)** and finally becoming the right pulmonary artery **(C)**. The aortic arch can be seen coursing over the right main stem bronchus, consistent with a right-sided aortic arch **(blue arrow)**.

Computed tomographic angiography (CTA) was performed to confirm the diagnosis and to further delineate the cardiac anatomy. CTA showed right atrial isomerism, pulmonary atresia and discontinuous branch PAs, right aortic arch with mirror image branching, and a vessel arising from the base of the innominate artery that supplied the left PA, with another vessel arising from the underside of the transverse aortic arch that supplied the right PA (Figure 1, Video 1). It also showed bilateral right bronchial anatomy and confirmed the presence of total anomalous pulmonary venous connection (TAPVC) with drainage into the left SVC–left-sided atrium junction without obstruction (Figure 2). In addition, the liver was noted to be midline, with the inferior vena cava draining into the right atrium and hepatic veins draining into the left atrium. A 3-dimensional model was printed to further understand the spatial orientation of this complex anatomy (Figures 3 and 4, Video 2) and for surgical planning. On the basis of the advanced imaging and 3-dimensional model, it was confirmed that the vessels feeding the PAs were bilateral patent ductus arteriosus on the basis of the typical location of the ducts in right aortic arch.

## Management

The patient underwent first-stage surgical repair with unifocalization of the branch PAs and placement of a modified Blalock-Thomas-Taussig shunt. Intraoperatively, the surgeons were able to confirm that the vessels in question had characteristics that were consistent with ductal vessels. Tissue from the vessel supplying the left PA was sent for histological examination, which identified it as ductal tissue.

## Discussion

The association between bilateral ductus arteriosus and pulmonary atresia has been recognized for decades. A study of 27 patients with bilateral ductus arteriosus done at the University of Toronto in 1984 revealed that 15 of those patients (55%) had pulmonary atresia with discontinuous PAs (1). Conversely, a study from Italy showed that among 247 patients diagnosed with pulmonary atresia, only 10 (4%) had bilateral ductus arteriosus (2). Interestingly, though, of those 10 patients with bilateral ductus arteriosus, 7 (70%) had right atrial isomerism. This points toward a significant association between bilateral ductus arteriosus and right atrial isomerism and shows that although most patients with bilateral ductus arteriosus have pulmonary atresia, only a minority of patients with pulmonary atresia have bilateral ductus arteriosus.

A review of the published research reveals that bilateral ductus arteriosus has been associated with several congenital heart diseases variants, most consisting of pulmonary atresia with ventricular septal defect. Indeed, to our knowledge and save 1 report (3), there has not been a documented case of bilateral ductus arteriosus with DORV. Our case is also novel because of the presence of right aortic arch and TAPVC.

Management of such cases initially relies on recognizing that the pulmonary circulation is entirely ductal dependent, and the use of prostaglandin E_2_ to maintain ductal patency is necessary. After initial stabilization, subsequent management strategies can focus on establishing more permanent pulmonary circulation. This can be done through stenting of bilateral ducti or though surgical intervention to establish stable pulmonary flow either through central shunts or through right ventricle–PA conduits. The presence of nonconfluent branch PAs adds several layers of complexity to establishing more stable pulmonary circulation. First, unifocalization of the discontinuous PAs will need to take place. Second, care must be taken to dissect all the ductal tissue to avoid constriction of ductal remnants after discontinuation of prostaglandin E_2_.

However, when pulmonary blood flow is supplied by aortopulmonary collateral vessels as opposed to ductal tissue, the strategy for surgical repair differs. The neonatal complete repair strategy, typically done between 3 and 6 months of age, involves early incorporation of as many aortopulmonary collateral vessels as possible into native PAs. Staged rehabilitation of native PAs with delayed complete repair is a viable alternative strategy.

## Follow-Up

The patient has done well post-operatively, with good weight gain and oxygen saturation between 72% and 91% on room air. She continues follow-up in our single-ventricle program.

## Conclusions

We present a rare case with a combination of right atrial isomerism, DORV with pulmonary atresia, and discontinuous branch PAs supplied by bilateral ducti from a right aortic arch with TAPVC to the left SVC–left atrium junction. This unusual anatomy highlights the intricate nature of congenital heart disease and the importance of advanced imaging such as CTA and 3-dimensional printed models in the setting of complex cardiac lesions for successful management.

## Funding Support and Author Disclosures

The authors have reported that they have no relationships relevant to the contents of this paper to disclose.

## Appendix

Supplemental Video 1 Aortic Arch. Rendering of the aortic arch from computed tomographic angiography showing the right ductus seen arising from the underside of the aortic arch and taking a tortuous course. The left ductus is seen originating from the undersurface of the left innominate artery and similarly assumes a tortuous course

Supplemental Video 2 Heart Model. Three-dimensional model of the heart and intrathoracic vessels. The video begins with an anterior view of the heart and intrathoracic vessels. It rotates to show the right-sided aortic arch, the origin and course of the right ductus (denoted by an asterisk at 00:05) as it supplies the right pulmonary artery, the origin and course of the left ductus (denoted by a caret at 00:16) as it supplies the left pulmonary artery, and the right and left superior venae cavae, shown in blue
